# Clonal Expansion of Infected CD4+ T Cells in People Living with HIV

**DOI:** 10.3390/v13102078

**Published:** 2021-10-15

**Authors:** John M. Coffin, Stephen H. Hughes

**Affiliations:** 1 Department of Molecular Biology and Microbiology, Tufts University, Boston, MA 02111, USA; john.coffin@tufts.edu; 2 HIV Dynamics and Replication Program, National Cancer Institute in Frederick, Frederick, MD 21702, USA

**Keywords:** HIV latency, HIV reservoir, HIV cure, persistent viremia

## Abstract

HIV infection is not curable with current antiretroviral therapy (ART) because a small fraction of CD4+ T cells infected prior to ART initiation persists. Understanding the nature of this latent reservoir and how it is created is essential to development of potentially curative strategies. The discovery that a large fraction of the persistently infected cells in individuals on suppressive ART are members of large clones greatly changed our view of the reservoir and how it arises. Rather than being the products of infection of resting cells, as was once thought, HIV persistence is largely or entirely a consequence of infection of cells that are either expanding or are destined to expand, primarily due to antigen-driven activation. Although most of the clones carry defective proviruses, some carry intact infectious proviruses; these clones comprise the majority of the reservoir. A large majority of both the defective and the intact infectious proviruses in clones of infected cells are transcriptionally silent; however, a small fraction expresses a few copies of unspliced HIV RNA. A much smaller fraction is responsible for production of low levels of infectious virus, which can rekindle infection when ART is stopped. Further understanding of the reservoir will be needed to clarify the mechanism(s) by which provirus expression is controlled in the clones of cells that constitute the reservoir.

## 1. Introduction

Combination antiretroviral therapy (ART), which was developed around 1997, leads to exponential decay of productively infected cells—measured by the level of virion RNA the infected cells release into blood—to below the limits of detection in the assays available at that time [1,2]. Extrapolation of the decay curve led to predictions that a relatively short course of ART would eradicate HIV-infected cells in infected individuals. However, when ART is discontinued, even in people in whom HIV infection has been fully suppressed for more than 10 years, the virus rapidly returns to pretherapy levels [3]. Although ART effectively blocks HIV replication in infected individuals, it does not cure them. ART almost always reduces viremia to levels undetectable by standard clinical assays; however, more sensitive assays show that there are very low levels of virus in the blood of the majority of treated individuals [4]. In ART treated individuals, rare CD4+ T cells (typically, 1–10 per million in samples from blood and lymphoid tissues) contain intact, infectious, viral DNA genomes (proviruses) that are not expressed in vivo. Some of these cells can be induced to produce virus particles ex vivo by treatment with one of a variety of agents known to induce T-cell activation, including phorbol myristic acetate (PMA) + ionomycin, anti-CD3, phytohemagglutinin (PHA), and others [5,6], collectively referred to as latency reversing agents (LRAs). Taken together, the cells that carry the intact infectious proviruses constitute a reservoir that persists for the life of all people living with HIV despite complete suppression of HIV replication [7]. A small fraction of the cells in the reservoir spontaneously releases infectious virus into the blood all of the time. The released virus rekindles systemic HIV infection if cART is discontinued. In most individuals on long-term cART, the number of cells containing inducible infectious provirus slowly declines with time on ART; however, the decline is too slow to eliminate the reservoir [8]. 

**Figure 1:**
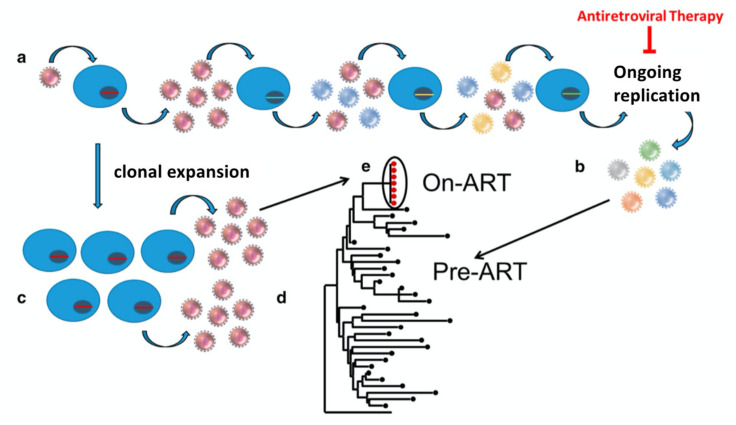
Ongoing HIV replication and clonal expansion. (**a**) In most individuals, HIV infection starts with a single virion (brown) and, during successive cycles of replication, shown left to right, the viruses in the population accumulate mutations, indicated in (**b**) by the multicolored virions, and, as shown in (**e**), a diverse phylogenetic structure. As indicated in (**c**), ART initiation halts ongoing viral replication, leaving a small fraction of infected cells, which, as shown in (**d**), grow into clones, a few of which can release virus with identical genome sequences into the blood. Reprinted from [10].

An early clue to the nature of the HIV reservoir was the observation that—in contrast to the highly diverse population of viral RNA genomes present prior to therapy—the low levels of virus found in blood during ART often included viruses whose genomes had identical sequences (Figure 1) [9]. It was initially unclear if these “predominant plasma clones” arose from isolated pockets of cells infected with single founder viruses (virus clones) or were the result of repeated division of cells derived from a single infected ancestor (clones of infected cells). We now know that, although a large majority of CD4+ T cells that are infected by HIV in vivo die within a few days of infection, a small fraction of the infected cells survive and divide, producing large clones, with important clinical consequences. The long-term persistence of the reservoir depends, to a significant extent, on the clonal expansion of cells infected prior to ART. 

In this review, we describe what has been learned about the clonal expansion of infected CD4+ T cells in vivo: when it occurs, how it occurs, and why it occurs. As we will discuss, the analysis is complicated by the fact that, in those on ART, most HIV-infected cells (including the cells in clones) carry defective copies of the HIV genome [11]. We will also consider the limitations inherent in the available data and will discuss some of the outstanding problems in the field; in particular the question of why it has been so difficult to cure HIV infection, and what is likely to be possible in the near future. It will be clear from reading this review that the integration of a DNA copy of the HIV genome into host DNA is a critical step in HIV replication, in the formation and persistence of the reservoir, and in pathogenesis [12,13,14,15]. 

## 2. Background: HIV Infection, Integration, and the Effects of ART on HIV Replication

HIV is a retrovirus, which means that the two identical single-stranded RNA genomes found in virions are converted, in newly infected cells, into a linear double-stranded DNA by the reverse transcriptase (RT) enzyme, which enters the cell in the virion. The viral DNA is identical in sequence organization to the viral genome, except for the presence of duplicated sequences at either end that form a structure known as the long terminal repeat (LTR). The linear viral DNA is inserted (integrated) into the genome of infected host cells by integrase (IN), which is also present in the virion. As a consequence, if an infected cell survives and divides, all of its descendants carry a provirus—an integrated DNA copy of the viral RNA genome. An HIV provirus resembles a normal host gene, having, within its LTRs, a promoter, binding sites for cellular transcription factors, a polyadenylation site, and internal splice donors and acceptors. Host DNA-dependent RNA polymerase produces full-length viral RNA from the integrated provirus. The full-length RNA serves both as genomic RNA and as mRNA from which polyproteins are made, which give rise to both viral structural proteins and enzymes. A complex collection of single and multiply spliced mRNAs is produced from some of the full-length viral RNA; these spliced messages give rise to additional viral proteins, including the Env surface glycoprotein and Tat and Rev which are necessary for efficient provirus transcription and nuclear export of viral RNAs.

By targeting the viral enzymes, ART effectively prevents viral replication but does not affect cells that are already infected, or the proviruses they carry. The host immune response and the toxicity associated with viral expression can lead to the rapid elimination of cells that are actively expressing HIV; however, cells that carry a provirus that is not expressed will not be eliminated. Although there have been claims that HIV persists in individuals who are on successful ART by limited viral replication in sanctuary sites [16], the available data strongly support the conclusion that there is no viral replication in individuals who are on successful ART [17]. The most direct evidence is that, in samples taken from both blood and lymph nodes of the majority of people living with HIV who are on successful ART, there is no further evolution of the virus [18,19] (Figure 1), a characteristic of chronic untreated HIV infection. In particular, HIV does not develop resistance to the drugs used in suppressive ART, even over decades of suppressive therapy. These data point to the long-term survival of HIV-infected cells as the primary reason that HIV infection is not cured by the currently available ART. The importance of the HIV provirus in permitting the infection to persist on ART is illustrated by comparing what happens when an individual with HIV is treated with successful ART with what happens when someone chronically infected with hepatitis C virus (HCV, a flavivirus that replicates through an RNA intermediate) is similarly treated with drugs that block its replication. Although, in untreated individuals, the HCV viral load is higher than the HIV viral load and HCV is more diverse than HIV, a 6-week treatment with drugs that effectively block HCV replication cures >95% of chronically infected individuals [20]. No one has been cured of HIV by ART alone.

The problem of persistence of HIV-infected cells arises from the fact that, as part of their normal role in the immune system, CD4+ T cells, the primary target for HIV infection in vivo, divide in response to their cognate antigens, then decline in the absence of antigen and the surviving cells are maintained by homeostatic signaling. Thus, it should not be surprising that, like their uninfected counterparts, HIV-infected CD4+ T cells that survive can also continue to divide in response to the same signals. In the resulting clones of infected cells, each of the daughter cells will have an identical provirus integrated in exactly the same place in its genome as its infected parent cell. Although only a tiny fraction (probably no more than 1 cell in 100,000) of the more than 10^8^ cells infected every day survives and forms clones prior to ART, this clonal expansion leads to the development of a large pool of infected cells. As will be discussed in more detail later, in some people living with HIV, clones of infected CD4+ T cells can grow to more than 10^8^ cells. In individuals on ART, the vast majority (95–98%) of the clones of infected cells carry defective proviruses [11,21]. However, the number of infected clones is large, and, in some individuals, there are large clones of infected cells that carry intact infectious proviruses [22,23]. At any given time, only a small fraction (5–10% or so) of cells in any one clone express viral RNA, and the measured levels of viral RNA appear to be too low to produce viral particles [24]. However, the persistent, low-level viremia seen in most people on ART implies that, at any given time, a very small fraction of the cells that carry an intact, infectious provirus are actively expressing viral RNA at levels sufficient to produce virions.

Curing HIV infection will require eliminating, or effectively and permanently silencing, all of the infectious proviruses, or permanently blocking replication of their progeny virus [25]. Eliminating all cells with intact proviruses poses a formidable challenge, since estimates suggest that their number is very large. If an infected individual has a normal level of CD4+ T cells, ca. 10^12^, if approximately one cell in a thousand is infected, and a few percent of those cells carry an infectious provirus, there will be on the order of 10^7^ to 10^8^ cells with intact proviruses. The number varies because, in people living with HIV, the number of surviving CD4+ T cells (CD4 count) varies, as does the fraction of infected CD4+ T cells. This grim picture is supported by the fact that, in HIV-infected individuals who underwent bone marrow transplantation as part of treatment for cancer, replacing more than 99% of their lymphocytes with cells from an HIV-susceptible donor greatly delayed the reappearance of virus, but did not cure their HIV infection [26,27]. Only in two cases in which such an allogeneic transplantation was performed with cells from a donor whose CD4+ T cells lacked CCR5, the primary co-receptor for HIV entry, have cures been reported [28,29]. The difficulty of obtaining a cure by allogeneic transplantation supports the idea that eliminating all the HIV-infected cells that can rekindle an infection when ART is discontinued will be a daunting task.

## 3. Integration Site Analysis

**Figure 2:**
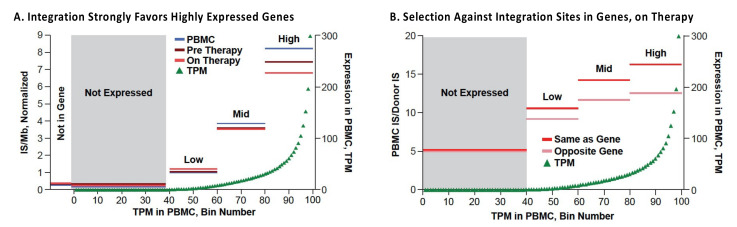
Distribution of HIV integration sites in genes in vitro and in vivo. The 22,000 protein- and long non-coding RNA genes in the hg19 database were divided into 100 bins based on the relative levels of the RNA in transcripts per million in PHA-stimulated donor PBMC (green triangles). (**A**) The bins were further combined into 4 groups (not-, low-, medium-, and high-expressed). The integration site (IS) density relative to total IS in PBMC infected in vitro (blue) and pre-ART (plum) and on-ART (red) donors was plotted for each expression group. (**B**) The on-ART IS data were further separated by orientation of the proviruses relative to the host gene and the data for the PBMC infected in vitro were divided the same way. Note that higher values indicate fewer integrations in the on-ART relative to the PBMC data. Reprinted from [37].

**Figure 3:**
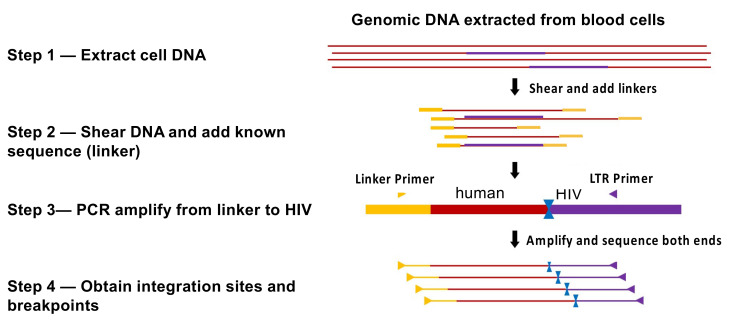
Integration site analysis. The method is based on an approach devised by Berry et al. [33]. DNA from infected cells is extracted and sheared to yield randomly distributed breakpoints in the host DNA, to which defined linkers are added. PCR amplification is initiated using primers complementary to the viral LTR and to the linker, ensuring that DNAs that have viral sequences are preferentially amplified. Amplification of DNAs with LTR and linker primers yields a collection of molecules with the integration site near one end and the breakpoint in the host DNA near the other. Both the sequence of the integration site and the breakpoint are determined by next-generation paired end sequencing. Clonally amplified proviruses are revealed by the presence of multiple sequences that have an identical integration site and that also have different breakpoints in the appended host DNA.

HIV preferentially integrates its DNA into the bodies of highly expressed genes in gene dense regions (Figure 2A) [30], with only a modest specificity for a target sequence [31]. Although integration is not random, there are millions of potential integration sites in the human genome. For that reason, the probability that two independently infected cells will have their proviruses integrated in exactly the same site is extremely small. Clones descended from one originally infected cell can be identified by the presence, in a sample (or samples) obtained from an individual, of multiple cells with identical proviruses at exactly the same integration site. Because viral replication can produce proviruses with identical sequences, the presence of proviruses with identical sequences is not proof that the proviruses are from a clone of expanded cells [32]. Thus, integration site analysis has been used to identify clones of HIV-infected cells. A number of methods have been used to identify integration sites; all rely on selective PCR, and most involve some form of linker-mediated (LM) PCR combined with next-generation sequencing. These methods all use isolated, purified, genomic DNA and, for that reason, are not affected by the structure of chromatin or any other aspect or state of the DNA in the host cell. One commonly used method [33] uses shearing of the DNA prior to LM-PCR and paired-end sequencing. The point of shearing is that the breakpoints in the host DNA provide unique sequence tags for each individual DNA molecule that is amplified by LM PCR. This approach makes it possible to estimate the relative number of different DNA molecules with the same integration site in a given sample, providing a measure of the extent of clonal expansion of the cells that carry a provirus integrated at that site (Figure 3). It is important to keep in mind that the count of the breakpoints associated with any one integration site does not depend on the numbers of times each amplicon was detected. Although it is possible, in rare cases, that breakpoints can be undercounted, MIDs (random sequences added to the linker) can be used to address that problem. Steps are also taken in the analytical pipeline to ensure that any breakpoints that arise artefactually are excluded. Further details of this and other methods, and their advantages and disadvantages, are beyond the scope of this review; the interested reader should consult the relevant literature [33,34,35]. It should be emphasized that, in people living with HIV, there is only one provirus in most infected CD4+ T cells, and only a small fraction of the CD4+ T cells carry a provirus. Therefore, identifying the integration sites from human donors is technically challenging, and a variety of artefacts can and do occur, including PCR artefacts such as mispriming and recombination [35,36]. It is not only important to be scrupulous in the wet-bench part of integration site analysis; it is at least as important to be rigorous and stringent in the accompanying informatic analysis of the integration site data.

## 4. Limitations of Integration Site Analysis

**Figure 4:**
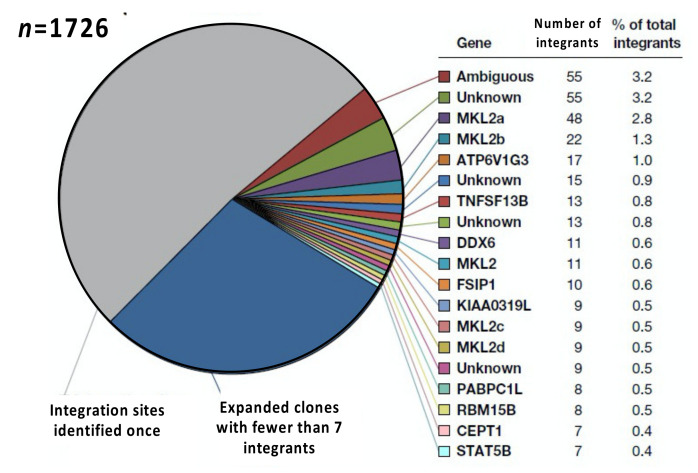
Clonal distribution of integration sites in one individual. The pie chart shows the integration sites obtained from Patient 1 reported by Maldarelli et al. [38], arranged by the relative sizes of the clones, as determined by recovering the same integration site linked to different breakpoints (shown as “number of integrants”). Sites with 7 or more breakpoints are colored and listed by gene on the right. Note that the largest clone, which is labelled “Ambiguous,” and whose provirus is referred to as “AMBI-1” in the text, is integrated in a sequence that is found in multiple locations, including a ribosomal RNA gene, of which there are many nearly identical copies. Data from Maldarelli et al. [38].

In addition to its power, the limitations of integration site analysis also need to be considered. The first important limitation is sampling. The efficiency of detection of integration sites in samples obtained from a human donor can be as high as 10% [38]; however, samples that can be obtained, processed, and analyzed represent only a tiny fraction of the infected cells in a person living with HIV. Of the more than 10^9^ infected CD4+ T cells in an infected individual on ART, only about 2% are in blood, and fewer than 0.2% of these are in a 10 mL blood sample. The remainder of the CD4+ T cells are in lymphoid and other tissues. Obtaining 10,000 integration sites from an individual donor requires both a generous donor and a major effort; if 1 cell in 1000 is infected, and if 10% of the integration sites are detected in a sample, obtaining 10,000 integration sites requires analysis of approximately 10^8^ CD4+ T cells, or about 600 µg of DNA, from at least 100 mL of blood. Since individual reactions typically use no more than 10 µg of DNA, very large numbers of replicates are needed to obtain this number of integration sites. With 10,000 integration sites, it is possible to detect clones of approximately 10^4^ cells reliably [18]. However, both sampling limitations and the efficiency of the recovery of integration sites means that it is not usually possible to obtain many more than this number of integration sites per assay from on-ART human donors. Those limitations mean that it is not possible to determine whether, in those on long-term ART, the large numbers of integration sites seen only once (i.e., those with only one breakpoint) represent large numbers of small clones of infected CD4+ T cells, large numbers of infected CD4+ T cells that have not clonally expanded, or both. In the example shown in Figure 4, of the 1726 integration sites detected about half were shown to be in clones. It is highly improbable that the clone size distribution drops off suddenly below the smallest detectable clone, so a large fraction of the “singles” must also be members of small clones. However, there is no good way to determine how large this fraction is. Given the data that are now available, neither the claims that nearly all the infected cells in those on ART have clonally expanded [39] nor the claims that there are “singles” (infected cells that have not clonally expanded) present in people on long-term ART [36] should be taken seriously.

Another issue is that, in its current form, large-scale integration site analysis does not provide useful information about the nature of a provirus at any particular integration site. Standard integration site assays do not distinguish between the sites of defective and intact, infectious proviruses, or the solo LTRs that can be formed by homologous recombination post-integration [40,41,42]. Techniques that make it possible to obtain both the structure of the provirus and its integration site have been developed. One method uses multiple displacement amplification of endpoint-diluted DNA to create multiple copies of genomic DNA containing a single integrated provirus, which can then be used for both integration site analysis and proviral sequencing [32,43,44]. At present, these methods are labor intensive, and the number of cases in which both the structure of a provirus and its cognate integration site are known is limited. This important constraint will be considered in more detail in a later section. Newer methods based on long-read sequencing technology have recently been reported [45], but have not yet been applied to on-ART samples from people living with HIV.

A third issue is that, although ca. 98% of CD4+ T cells, both infected and uninfected, are in lymphoid and other tissues, tissue samples of sufficient size are much more difficult to obtain from living human donors, and tissue samples often have much lower frequencies of HIV proviruses than blood samples. Therefore, most, but not all, in vivo integration site analysis has been performed with samples obtained from blood. Relatively little is known about the clonal expansion of HIV-infected cells in the tissues of individuals who are on ART; even less is known about the behavior of clones of HIV-infected cells in the tissues of individuals who are not on ART. There are some data from lymph node biopsies taken from living donors [19], and a limited amount of data from non-lymphoid tissues have been obtained from autopsy samples [46]. There are also limited data comparing the distribution of clones of SIV infected cells in blood and tissue samples obtained from SIV-infected macaques [47]. Although the amount of useful integration site data from solid tissues is still relatively small, it is clear that in both people living with HIV and in SIV-infected macaques on long-term ART, some large clones are widely distributed in the blood and in multiple lymph nodes and other tissues [46,47]. Although the analyses that have been reported provide no evidence for strict compartmentalization of infected cell clones between blood and tissues, it is likely that some individual clones of infected CD4+ T cells have distinct homing properties based on their subset and antigen specificity. Uninfected T cells are preferentially found in tissues that express the antigen that they recognize. Similarly, clones of infected T cells may also be preferentially found in tissues that express an antigen that they recognize. In particular, pathogens (such as HIV and CMV) that cells in HIV infected clones have been shown to recognize are not equally distributed in all tissues. In addition, clones of infected cells may vary in the rate at which they traffic in and out of blood, lymph, and tissues, reflecting differential expression of— and response to—homing ligands and receptors. In the case of AMBI-1, for example, the largest known clone with an intact, infectious provirus (“ambiguous” in the example shown in Figure 4), the size of the clone varied with the growth and treatment of a cancer in the donor. Further, the cells that carried the AMBI-1 provirus were significantly enriched in the tumor-infiltrating T cells [23], suggesting that the cells in this clone may have been responsive to a tumor-specific antigen or some other tumor-specific factor. However, both the AMBI-1-infected cells and the progeny virions were also found in the blood plasma [23]. Some of the plasma virion RNA may have come from virus produced in other lymphoid tissue. Much more work needs to be undertaken to understand how specific clones of infected cells are distributed in blood and tissues and how that distribution affects the distribution of virions.

There does not yet appear to be any reason to think that the rules must necessarily be the same for all of the clones of infected cells. If a clone of infected cells carries a highly defective or non-expressed provirus (i.e., is unlikely to express a viral antigen), the integrated provirus is, in most cases, unlikely to perturb the behavior of the infected cells. Important but rare exceptions are discussed below. In cases in which the provirus does not affect the behavior of the host cell, the integration site of the provirus provides a convenient marker that is easier to follow than the T cell receptor (TCR), and the integration site can be easily used to identify the cells of that clone and track their behavior [48].

Finally, if a provirus is integrated in highly repeated DNA, the currently available methods using short-read technology cannot readily distinguish among the different copies of the highly repeated DNA. This limitation restricts many of the conclusions that can be reached to proviruses integrated in or very near single-copy regions of the human genome. Fortunately, as will be discussed in the next section, HIV preferentially integrates in the bodies of highly expressed genes, most of which are single-copy. Determining whether proviruses integrated in a particular gene or region of the human genome are selected for or against requires a careful comparison of the numbers/density of the proviruses found in that gene or region immediately after the initial infection, and after a long enough time on ART so that any selection against cells with expressed proviruses would have changed the initial distribution. For this reason, accurate quantitation and precise localization of proviruses integrated in centromeres [49], LINE elements, and other highly repeated sequences will have to wait for a better way to enumerate the proviruses integrated into repeated regions of the human genome. 

Despite these limitations, integration site analysis has provided a great deal of useful information about the formation, nature, and persistence of HIV-infected cells, about the reservoir, and why it has been impossible to cure HIV infections with available ART. Clones are present pre-ART [18], but are much more easily detected on ART, because after ART is initiated, most of the recently infected cells die with a half-life of 1–2 days [50]. Thus, it is important to remember that clones of HIV-infected cells are not created by ART; they are revealed by ART. Despite the limitations in the number of integration sites that can be obtained, at least half of the proviruses in the blood of those on ART are in clonally expanded cells. Despite the fact that most clones carry defective proviruses, large clones can also carry infectious proviruses, and can make up, at the least, a significant part of the reservoir [22,23]. 

## 5. Distribution of HIV Integration Sites

**Figure 5:**
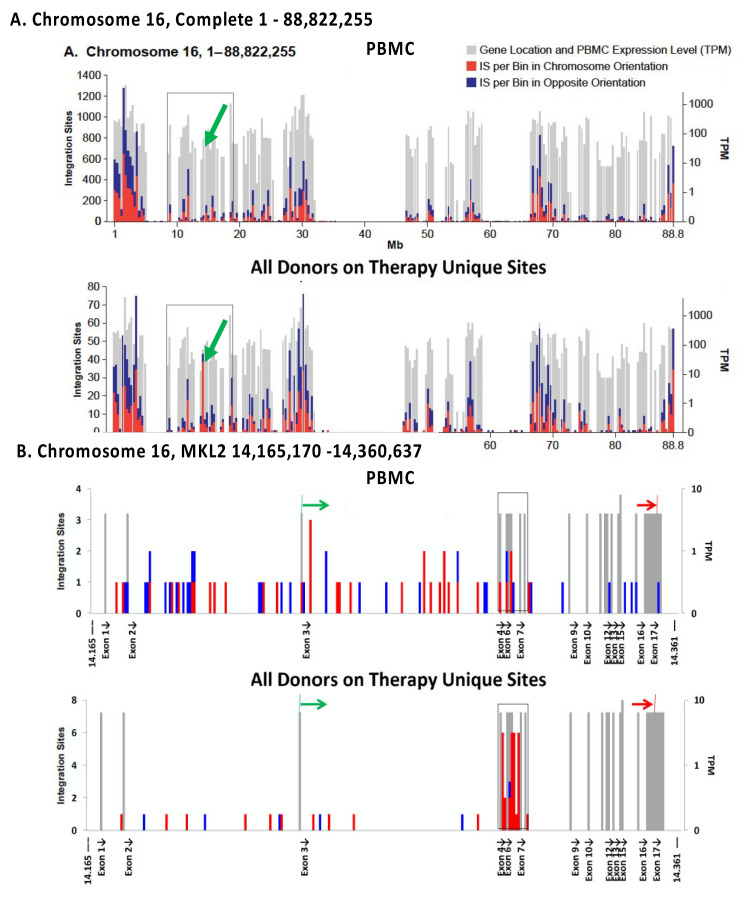
Integration site distribution in vivo and in vitro. Chromosome 16 was divided into 250 bins and the level of RNA, as measured by RNA-seq analysis of PHA-activated PBMCs, in each bin (gray bars) and the integration site distribution (red—the provirus is in the same orientation as the numbering of the chromosome; blue—opposite orientation, relative to the numbering of the chromosome) are shown for each bin. In both panels (**A**,**B**), the diagram at the top shows data obtained from PBMCs infected in vitro; the diagram at the bottom shows unique sites from pooled in vivo data. (**A**) Complete chromosome 16. The long green arrows indicate the location of the *MKL2* gene. (**B**) *MKL2* gene. Black arrows indicate exons; short green and red arrows, translation start and stop sites, respectively. Reprinted from [37].

The in vivo distribution of HIV DNA integration sites in those on ART is largely determined by the initial distribution of sites, as defined by ex vivo analysis of freshly infected PBMC (Figure 2A) [37]. It has been known for some time that HIV proviruses are preferentially found in highly expressed genes in gene-rich regions [30]. This preference is primarily due to interactions of the capsid (CA) of the HIV virion with the host factor CPSF6 [51], which mediates passage through the nuclear pore [52] and the viral integrase protein with LEDGF/p75, a chromatin binding factor that is associated with the bodies of actively transcribed genes [53,54,55]. The overall distribution of HIV proviruses can be subsequently modified by selection that favors or disfavors the survival of cells that have proviruses integrated into a particular gene or region of the host genome. Comparison of the distribution of the HIV integration sites in PBMC infected in culture and cells taken from those living with HIV, both before and after ART, showed that, although the overall distributions of the integration sites were quite similar (Figure 5A), there are some modest but important differences [37]. In vivo modifications of the proviral distribution can be divided into three categories: negative selection against cells with proviruses in genes; positive selection for proviruses in a few specific genes, and clonal expansion of infected cells independent of presence of a provirus.

Negative selection against cells containing proviruses that are integrated in the bodies of highly expressed genes is seen in individuals on long-term ART (Figure 2B) [37]. The strength of this selection correlates with the level of expression of the host gene; there is no detectable selection against proviruses not in genes, or integrated in genes that are not expressed in CD4+ T cells. In addition, selection is stronger against proviruses in the same orientation as their host genes, compared to proviruses that are integrated in the opposite orientation. The selection is both broad (in that it involves many genes) and is relatively weak for any particular gene. The simplest interpretation of the data is that this negative selection is not related to the expression of the provirus but is rather a result of the effects the integrated proviruses have on the expression of the host genes in which they reside—most likely due to disruption of expression and/or processing of the RNA for the host gene by proviral splicing and polyadenylation signals. This explanation accounts for the observation that selection against proviruses in the same orientation as the host gene is stronger than selection against proviruses in the opposite orientation. It also explains why the negative selection is stronger for proviruses integrated in genes that are expressed at a high level and the observation that the effects are both broad and deep. 

The most striking difference in the distribution of integration sites in vivo and ex vivo is presence, in samples taken from those on long-term ART, of clusters of proviruses in a few specific introns in a handful of oncogenes (Figure 5) [38,56]. We recently identified seven such genes in which a provirus can cause positive selection for the growth and/or survival of the host cell (Table 1) [37]. While it is possible that a much deeper analysis might reveal a few more such clusters, the number of genes in which a provirus can provide positive selection for the host cell will remain very small. Although the regions defined by these clusters have sometimes been referred to as “hot spots” for proviral integration, with the exception of the first intron of *STAT5B*, they are not particularly good targets for integration. In addition, despite the fact that there is no evidence for integration of proviruses in a specific orientation in any gene, either in vivo or in vitro, the selected proviruses are always found in the same transcriptional orientation as the host gene. These observations strongly support the conclusion that the clustering is the consequence of post-integration selection acting on cells that happened to have acquired a provirus in the right place and orientation.

## 6. Selection for Proviruses Integrated in Certain Genes 

**Figure 6:**
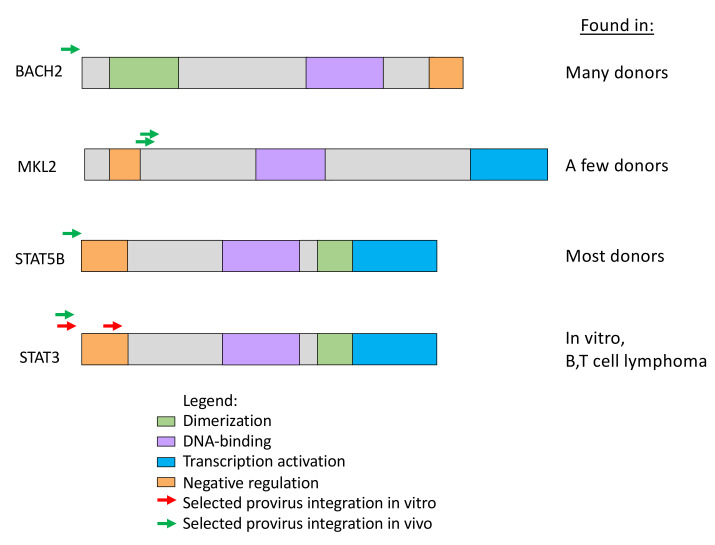
Proviruses in clonally expanded cells can affect the expression of the host gene. The schematic domain organization of the encoded protein products for four genes in which an HIV provirus can provide a selective advantage to the host cell are shown by the colored boxes. Red arrows indicate the orientation and location of selected HIV proviruses in vivo. Note that the *STAT3* integration sites are seen amplified only in vitro [58] and in AIDS lymphomas [59]. Provided by Machika Kaku.

**Figure 7:**
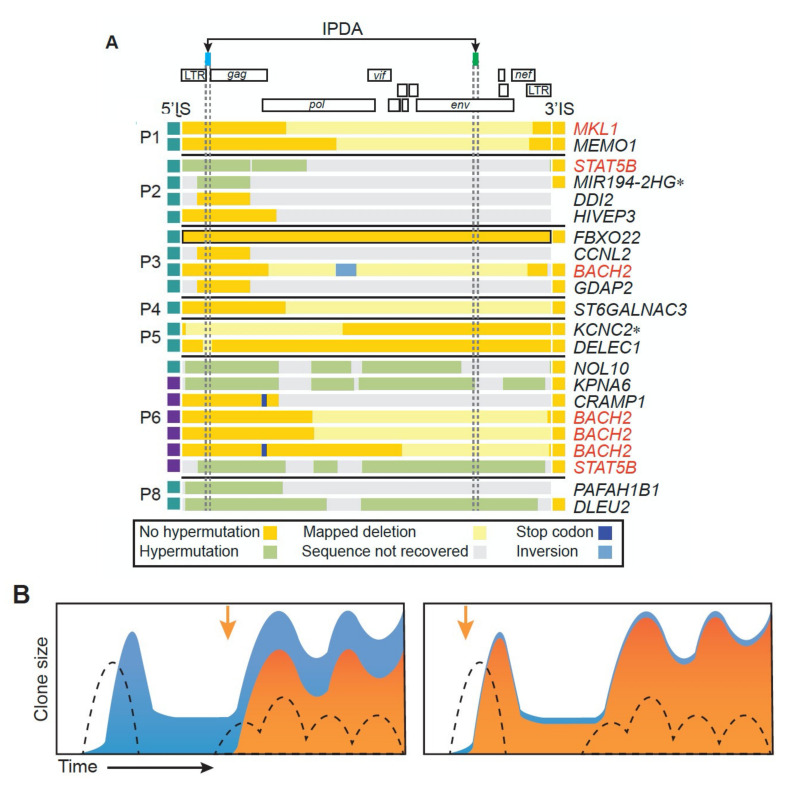
Antigen-driven clonal expansion. (**A**) Structures of proviruses reported by Simonetti et al. [60] in clonally expanded cells obtained from the on-ART donors indicated on the left are shown schematically, along with the gene in which the provirus was integrated. Genes shown in red are ones in which proviruses can confer a selective advantage (Table 1). The 2 regions marked “IPDA” have been found to be commonly deleted or hypermutated in defective proviruses and are the basis for the selective Intact Provirus Detection Assay [61]. CD4+ T cell clones containing the proviruses shown were reactive to one of two common antigens associated with HIV infection, as shown in the boxes to the left of the provirus: CMV (teal), or HIV Gag (purple). (**B**) Schematic showing the importance of the relative timing of antigen stimulation and integration on the frequency of cells in a specific HIV-infected T-cell clone. The dotted lines show the relative concentration of stimulating antigen (CMV or HIV, in this case). Solid blue indicates the total size of the specific antigen-reactive CD4+ T cell clone, and the solid orange indicates the proportion of cells in that clone that carry a provirus descended from a cell infected at the time shown by the orange arrow. Note that the time of infection relative to the origin of the CD4+ T cell clone is reflected by the fraction of the cells with a specific rearranged T cell receptor that contain a provirus at a specific integration site. Copyright 2021 American Society for Clinical Investigation (ASCI).

What might be the mechanism of this in vivo selection? All the targeted genes belong to the very large set of genes whose expression, or, more accurately, misexpression, is associated with cancer. Importantly, although there are hundreds of such cancer-associated genes, many of which are good targets for HIV integration, no other gene shows the same pattern of clustered integration sites in vivo and not in vitro as these seven genes. Thus, whatever feature(s) these genes share that leads to selection of cells with proviruses in them, it is not simply the fact of their being cancer associated. In addition, the proviruses in the selected cells are positioned in a way that could modify the expression of the protein product (or, in some cases, of a truncated version of the protein) (Figure 6), a feature which is strongly reminiscent of the well-studied models for cancer in experimental animals, particularly chickens and mice, by alpha-, beta-, and gammaretroviruses [57]. But these HIV-infected cells are not cancer cells. Although the insertional modification of gene expression has provided the cells with some selective advantage, it has not led to their uncontrolled growth. It is possible, then, that misexpression of these genes does not bypass normal signaling, but rather makes the cell more sensitive to growth promotion by them. Alternatively, since memory CD4+ T cells, the principal targets for HIV infection, normally experience periods of rapid expansion when cognate antigen is presented, followed by contraction by apoptosis in the absence of antigen (Figure 7B), misexpression of genes could reduce the cell’s sensitivity to proapoptotic signaling, which could provide a selective advantage by allowing preferential survival of the cells during the contraction phase. Whatever the mechanism, selection for cells with proviruses in these genes is unlikely to be an important mechanism of HIV persistence, because, to date, all of the proviruses seen in these genes were found to be highly defective (for example, see Figure 7A).

**Figure 8:**
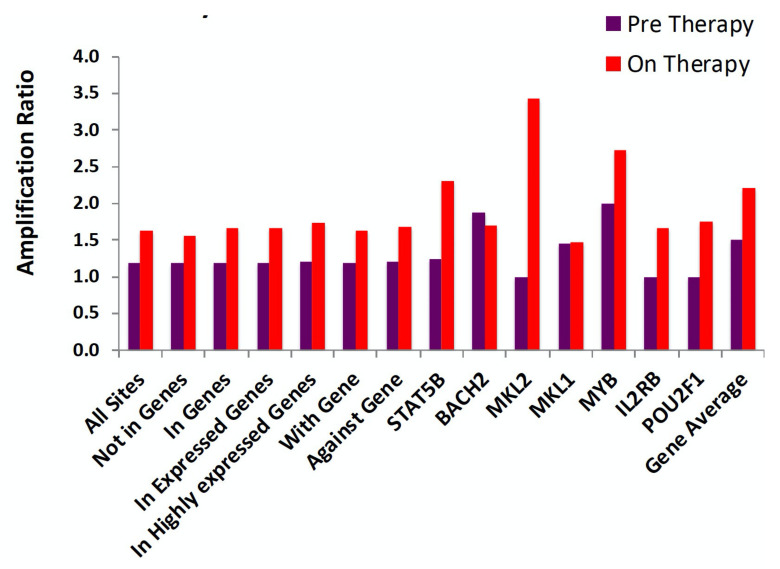
Clonal amplification is independent of integration site location or provirus orientation. Pooled integration site data from pre- and on-ART samples were divided into the groups shown, and the amplification ratio was calculated using the total number of breakpoints in each group divided by the total number of unique integration sites. None of the differences are statistically significant. Reprinted from [37].

Despite being a striking effect, the overall impact of proviruses integrated in these seven oncogenes, taken together, is not large, amounting to only a few percent of the infected cells, and the cells that carry them do not show significantly higher levels of clonal amplification than the average for all of the infected CD4+ T cells (Figure 8) [57]. These observations suggest that immunological mechanisms are the primary drivers of clonal expansion. Such mechanisms would be expected to act independently of the location or orientation of a provirus, consistent with the results shown in Figure 8. Two mechanisms of immunologically driven clonal expansion of HIV-infected CD4+ T cells have been proposed: division in response to stimulation with (1) a cognate antigen, or (2) with homeostatic cytokines, such as IL-7. The relative contribution of the two mechanisms is not known. While it is very clear that at least a fraction of the highly expanded clones are responsive to a specific antigen, such as Cytomegalovirus (CMV) and HIV itself [60], the importance of homeostatic signaling to the process is unclear. It seems unlikely that even the smallest identifiable clones, which are larger than 10^4^ cells, would have been lucky enough to have responded repeatedly to random homeostatic signaling for the 15 or more generations needed to reach that size. 

## 7. Infected T Cells That Give Rise to Clones of Infected T Cells

A recent study by Simonetti et al. [60], in which the authors were able to identify the integration site of the provirus, the TCR rearrangement, and the specific stimulating antigen (HIV Gag or CMV) for a number of highly expanded clones, has shed additional light on the relationship between proviral- and antigen-driven expansion. Using PCR assays based on the integration site and TCR sequence, they showed that some of the clones were infected at or near the time of initiation of clonal outgrowth, and others long after the clone had started to grow (Figure 7B). They also made the intriguing observation that, in more than one-third (7/22) of the antigen-driven clones they studied, the provirus was integrated within one of the seven genes associated with selection of infected cells. This frequency is much greater than that observed in unselected cells in another cohort [37]. While much more work is needed, the suggestion of a more than 10-fold greater frequency of proviruses in one of the seven genes in large clones selected for antigen reactivity seems to imply some sort of synergy between clonal selection by an antigen and the effects of provirus-driven gene misexpression. However, if such a synergistic mechanism does operate, its impact must be limited, or the faction of infected cells with a provirus in the seven genes would be considerably higher than it is (a few percent). 

With the exception of the work of Simonetti et al. [60], which was just discussed, relatively little is known that helps us understand the nature of the initial infected cells that can give rise to large clones. It was recently proposed that the infection of naïve T cells is important in generating HIV-infected clones [62]. While it is not possible to rule out this possibility, we do not think it is a major pathway that leads to the generation of clones of infected cells. Naïve T cells do not express the CCR5 coreceptor. In most individuals on ART, the majority of the proviruses encode an Env that uses CCR5. In addition, most of the viruses that emerge in vivo when therapy is stopped, or in vitro when cells are stimulated to produce virus, use CCR5. These data suggest that the majority of the initially infected cells that give rise to the reservoir express CCR5. There is an older proposal that the reservoir is formed primarily by the infection of T cells that are about to transition into quiescent long-term memory cells [63]. Although that sort of event may occur, it is not likely that it makes a major contribution to the generation of the reservoir. First, we now believe that clonally expanded cells make up a significant fraction (perhaps all) of the reservoir. A recently infected T cell that is about to transition in a quiescent long-term memory cell is not likely to form a large clone. Second, dividing cells make much better targets for HIV infection than cells that have stopped dividing. Finally, the data of Simonetti et al. [60] show that, in some cases, clones of infected cells arise by the infection of a cell in a clone of antigen-specific uninfected cells that then continues to divide so that only a fraction of cells with identical T-cell receptor rearrangements carry a provirus at a specific integration site.

At this time, it is unclear whether the mechanisms of clonal expansion apply equally to clones that carry intact infectious or defective proviruses. From the few observations available, it is clear that cells that carry infectious proviruses can expand before and during ART into large clones that produce drug-sensitive virus at detectable levels. In such cases, viremia cannot be suppressed by changing the ART regimen, as would be expected if the virus is produced by a large clone, is not the product of ongoing replication, and the viruses are not drug resistant [4,21]. As mentioned earlier, most of the available data on the clonal expansion of infected CD4+ T cells do not distinguish between clones of infected cells that carry intact infectious proviruses and clones that carry defective proviruses. Possible differences in the behavior of clones of infected cells that do and do not carry intact infectious proviruses will be considered in more detail later. Although the data that are available thus far are quite limited, all of the proviruses in the seven oncogenes that have been characterized to date have been defective, as were all the proviruses described by Simonetti et al. [60] (Figure 7A). Given that intact proviruses constitute only about 2–5% of the total [37], a much larger study will be needed to exclude the possibility that there are rare clones of infected CD4+ T cells in which an intact infectious provirus is able to contribute to clonal expansion by enhancing the expression of one of the seven oncogenes. 

It was recently reported that, in people on long-term ART, the fraction of intact proviruses integrated in genes was lower than the fraction of defective proviruses integrated in genes [44]. This result was interpreted to imply that integration in genes somehow favors expression of the provirus, leading to negative selection against cells with intact proviruses integrated in genes. However, the reported effect was statistically marginal, and, as has been discussed, there are a number of counterexamples of clonally expanded cells containing intact proviruses expressed at levels sufficient to give rise to detectable viremia [22,38]. There is a related report that in elite controllers—rare individuals living with HIV who naturally maintain very low levels of viremia without ART—intact proviruses were preferentially found in highly repetitive centromeric repeat DNA. Elite control is most likely due to a particular combination of MHC type and viral sequence that creates an especially potent T cell response from which the virus is unable to escape by simple mutation(s) [64]. Because accurate quantitation of proviruses integrated in repetitive DNA is not yet possible, the claim needs to be confirmed when methods that yield longer DNA sequences at integration sites are available and allow better quantitation of the fraction of the intact proviruses in repetitive DNA. As discussed above, there is also the problem of whether proviruses integrated in specific regions, including centromeric repeats are, or are not, expressed [49]. 

Thus, the major determinant in the overall distribution of proviruses in those on long-term ART is the initial distribution when the cells were initially infected, modestly modified by both positive and negative selection. This is a remarkable outcome, given that only a tiny fraction of the infected cells survive and form the clones that are present in people on long-term ART.

## 8. Proviral Expression Affects the Survival of Infected Cells and Clonal Expansion

The rapid and preferential loss of infected cells that are making virus following initiation of ART, as measured by a decline in the level of viremia, is a result of the rapid death of previously infected, virus-producing cells due to the toxicity of viral expression and the immune response of the host [50]. During fully suppressive ART, both types of selection will favor the long-term survival of cells that carry silent or highly defective proviruses; however, the problem is more complex than it initially appears. First, it has been proposed that deleted proviruses can express viral antigens [65]. Second, although the available data are still limited, it appears that only a small fraction of the cells in clones in individuals on ART express viral RNA, whether they carry intact or defective proviruses [24]. In most cases, the expression of viral RNA is at levels that are too low to support virus production, even if the provirus is intact. It is likely that one of the reasons that some clones are able to survive and proliferate is that only a small fraction of the cells in the clones express viral RNA, and, by extension, viral antigens, at any one time. This point will be discussed in more detail later.

The rapid and profound loss of infected cells that express high levels of HIV in those who initiate ART does not have a large effect on the overall distribution of proviruses [37]. This observation implies that, although expression of the proviruses in the cells that survive (and their descendants) must be strongly suppressed, the location of a provirus in the genome does not have a large effect on suppression. If there were genes, or regions of the genome, in which HIV proviruses were either much more likely to be expressed, or much less likely to be expressed, selection against cells that express viral proteins would affect the distribution of the proviruses in those on ART. The idea that the position at which an HIV provirus is integrated does not have a profound effect on its expression is supported by in vitro experiments performed with cells infected with HIV vectors [66]. It is helpful to think about the issue of whether, and to what extent, the integration site of a provirus is likely to affect the expression of the provirus based on what would be advantageous for the virus. A provirus that can be expressed at a high level wherever it is integrated will have an obvious advantage over a provirus that can be efficiently expressed in only a fraction of its integration sites. Thus, it is likely that the structure of the HIV provirus was selected so that it would be expressed efficiently if it was integrated in essentially any location in the human genome. 

## 9. Timing of Clonal Expansion of HIV-Infected Cells

Individuals that are started on suppressive ART within the first few weeks following HIV infection almost always exhibit virus rebound even if ART is discontinued after more than 2 years of therapy [67]. That result shows that the reservoir, cells that are capable releasing infectious virus, arises very soon after virus acquisition. If clonal expansion of HIV-infected cells plays a major role in the formation of the reservoir, clones of infected cells should appear shortly after the initial infection. The ability to determine exactly when clones first arise is limited by the sensitivity of detection of clones (clones must contain ca. 10^4^ to 10^5^ cells to be detected reliably). Generating a clone of 10^5^ cells requires about 16 cell divisions, assuming no loss of cells. In one study, multiple clones of at least 10^5^ cells were detected about 4 weeks after an initial infection [18]. These clones must have arisen from cells infected no later than 2–3 weeks following the initial infection of the individual, depending on the doubling time of the infected cells. Some of the clones detected in the first few weeks persisted for years on therapy, and clones were detected in all samples tested after several years on therapy, implying that they were already present, but too small to detect, as early as 3 weeks following the initial infection event. The rapid appearance of the clones is not consistent with the idea that infected cells that persist arise from infection of some kind of “resting” cell [68]. Thus, cells that give rise to clones must be infected, and dividing rapidly, shortly after a person is initially infected, consistent with reactivity to some antigen such as HIV Gag [60], present at the time. 

It is likely that the rare cells that gave rise to clones are infected, and the clones generate, as a more-or-less constant fraction of infected cells during the entire pre-ART infection period, when the virus is replicating freely. However, there are data that suggest, in at least some individuals who were infected with HIV for some time before going on ART, that an unexpectedly large fraction of the clones found on long-term ART were derived from cells infected shortly before ART was initiated [69]. Although there are several possible explanations for this observation, the simplest model is based on the fact that infected cells that do not express viral RNA likely remain susceptible to infection. Although the overall frequency of HIV infection of CD4+ T cells is low—less than 1% in chronic infection—the cells that survive an infection (or their descendants) are not likely to carry an expressed provirus and are likely to remain highly susceptible to infection. Therefore, before ART is initiated, it is likely that many of the infected cells carrying defective and/or silent proviruses that would have been able to form clones are killed by superinfection. However, when a person living with HIV is put on ART, new rounds of HIV infection are blocked. Thus, ART protects any recently formed clones of infected CD4+ T cells, or infected cells that could give rise to clones, from superinfection, which would, in the absence of ART, have killed the cells. Thus, a larger fraction of the cells infected shortly before ART was initiated can survive and grow to form clones. In cases where clones have expanded to detectable size pre-ART, some genetic or epigenetic feature may be protecting the cells in the clones from superinfection.

## 10. What Regulates Proviral Expression in Clonally Amplified Cells?

This question leads directly to a much larger question: what causes proviral latency in vivo? There is a simple answer: we have no idea. Although a full discussion of the larger question is well beyond the scope of the present review, we will make a few general points here. A number of in vitro models of latency have been developed [70], most of which involve cells infected with HIV vectors expressing a fluorescent marker and selected for loss of expression. It is not known whether any of these models accurately reflect latency in vivo. First, the fundamental issue in trying to develop an in vitro model is that we know so little about the properties of latently HIV-infected cells in vivo. Although in vitro models have value in that they can be used to study, and better understand, gene regulation, and to help identify some of the factors that can affect expression from the HIV promoter, how can we know how good an in vitro model is if we have no idea what the model is supposed to recapitulate? As we will discuss in more detail below, we do not know if the same factors control latency in all of the clones of infected cells in vivo, or if the same rules apply to clones that carry intact infectious and defective proviruses. 

To give a specific example that illustrates the underlying problem: in at least one widely used in vitro model, the latent proviruses were found to be extensively methylated in cytosine residues [71], a well-known mechanism that leads to the suppression of gene expression in other systems, notably in endogenous retroviruses [72]. By contrast, studies have failed to detect methylation of the LTR promoter above experimental background in both defective and intact infectious proviruses in people living with HIV who were on long-term ART [73,74]. As has already been discussed, the possibility that integration of proviruses in certain regions of the genome is incompatible with their high-level expression [44], with the possible exception of integration in centromeric repeats [49], is not supported by either in vivo [37] or in vitro [66] data. A third possibility is that the Tat-Rev regulatory circuit leads to stochastic on–off “flickering” of LTR-driven expression, eventually settling into either a (more or less) permanently active or silent state, which is only rarely reversed [75,76]. In that context, it is noteworthy that a large fraction of the inactivating deletions in defective provirus involve either the major splice donor upstream of *gag* or the region where *tat* and *rev* overlap *env* [61]. Both types of mutation would be expected to block high-level expression of all viral genes, even ones with intact reading frames, but might still allow occasional low-level Tat-independent transcription of the entire provirus. Finally, it was proposed that entry of an infected cell into a resting state shortly after infection might be associated with downregulation of transcription factors necessary for proviral expression [77]. The findings, discussed above, that large clones of infected cells develop rapidly after an individual is infected and that the large majority of the infected cells have expanded in response to antigen stimulation seem to argue strongly against this model, although HIV latency is still sometimes referred to as a phenomenon that occurs primarily in resting cells [78]. The rarity with which an initially infected cell becomes a reservoir cell, harboring a latent provirus, leaves open the possibility that there is something different, either genetically or epigenetically, in the host cell that enables the provirus to become latent. The rarity of these cells, especially those with potentially infectious proviruses, also makes their isolation and analysis, which is essential to an understanding of the mechanisms of latency, extremely difficult.

The clonal structure of the population of latently infected cells provides some clues into mechanism both of suppression of viral expression and of viral rebound, although much work remains to be done. Analysis of individual infected cells from people living with HIV during suppressive ART using a highly sensitive assay for unspliced (*gag-pro-pol*) RNA, showed that about 90–95% of the infected cells did not express detectable levels of viral RNA whether the provirus was intact or defective. A similar large fraction of proviruses was not expressed in both the bulk population of infected cells and in individual clones. The remaining cells expressed viral RNA at very low levels (ca 1–5 copies/cell) [24]. High levels (>20 copies/cell) of *gag-pro-pol* RNA were seen only in a small fraction of cells pre-ART [79]. It is the cells with high levels of viral RNA that are likely to be making virus. The failure to detect cells that make high levels of viral RNA in those on ART is not surprising because the very low levels of viremia in people on ART suggests that virus-producing cells are quite rare in these individuals. Persistent low level viremia, the presence of rare high-level virus RNA positive cells in lymphoid tissue in SIV models [80], and the clonal nature of the persistent virus [7,9], taken together, imply that the low levels of virus present in the blood of those on ART are released by a small fraction of the cells from a small number of clones. This persistent virus is very likely to be a source of rebound viremia when ART is discontinued [81,82]. Understanding the origin of the virus that causes rebound viremia would be an important step toward developing a curative strategy for HIV infection.

In a few cases, for example in the AMBI-1 clone discussed above (Figure 4), some cells within a clone can produce sufficient amounts of virus to be detectable by routine clinical assays [23]. The integration site for AMBI-1 is found in multiple locations, including in a ribosomal RNA (rRNA) gene cluster. Single cell analysis showed that out of more than 1000 peripheral blood cells analyzed, only about 2.3% contained any AMBI-1 *gag-pro-pol* RNA, and those cells contained an average of 2.5 copies of viral RNA/cell, which would be far too low to produce virions [24]. While this is a single case, it illustrates the magnitude of the problem. At the time of sampling, this individual had clinically detectable viremia, derived almost entirely from the AMBI-1 provirus, in excess of 100 copies RNA/mL. Yet, although it was the most highly expanded clone, AMBI-1 represented only about 3% of the total infected cells. Because such a small fraction of the AMB-1-containing cells expressed virus RNA, the virus producing cells represented less than about 3 per 100,000 of all infected cells. Extrapolation to the more typical case, persistent viremia of three copies/mL, would imply that the virus likely to account for rebound viremia is produced by less than one in a million infected cells. Since only about 1 in 1000, or fewer, CD4+ T cells in individuals on ART contain a provirus, this proportion would correspond to about 1 in a billion CD4+ T cells, or about a thousand cells total, which could reside in any site in the body from which the virus could be shed into the blood. These daunting numbers define the challenge of determining cause(s) of the re-emergence of an active infection following ART discontinuation.

Of the several approaches that are being tested to try to eradicate HIV infection, one, “shock and kill,” relies on using one or a combination of a variety of LRAs to induce expression of latent proviruses during ART. If high-level HIV expression could be induced, it should then lead to death of the infected cells, either directly or via immune-mediated killing [83]. A number of LRA strategies have been found to be modestly effective at increasing the levels of infectious HIV both in in vitro latency models and in isolated T cells from individuals on suppressive therapy. While newer, more potent approaches are under development, some basic questions regarding proviral latency need to be answered.

First, within a clone of cells with identical proviruses at the same integration site, why are the proviruses in some cells expressed at either a low or high level, while the proviruses in most of the cells are not expressed? Could this differential expression be due to the sort of stochastic flickering of expression mentioned above, to differential exposure of the cells to external signals, to different stages of differentiation of the cells (or transition between stages), or to different stages of the cell cycle?

Second, are the mechanisms regulating expression of the provirus the same for all clones? Importantly, are the rules that govern expression the same for cells with intact or defective proviruses? Because 95–98% of total proviruses are defective [11,21], cells containing defective proviruses dominate bulk DNA and RNA assays. Although there are similarities in both fraction of cells that express RNA from defective and intact proviruses and the level of expression of the defective and intact infectious proviruses, the implied similarity in the mechanisms that regulate the expression of the proviruses may be misleading. As has already been discussed, a large majority of clones carry defective proviruses that have deletions that affect either or both the major splice donor 5′ of *gag* or the *tat* and *rev* genes. These proviruses would be incapable of expressing Tat and Rev, and thus of high-level expression of viral RNA, whether they do or do not encode intact viral proteins. By contrast, cells that carry intact, infectious proviruses, with a functional *tat-rev* system, must use some other mechanism to control the expression of their proviruses. Thus, in vivo studies of LRAs that measured bulk proviral DNA as a readout could have been falsely reported as negative even if all the intact proviruses were eliminated, which would not have noticeably affected the total provirus load. Methods such as the intact proviral detection assay (IPDA), which targets the two most frequently deleted (or hypermutated) regions in defective proviruses (Figure 7A) have been designed to partly overcome this problem [61], but are not yet a perfect measure of the reservoir [84].

Third, we do not really know how LRAs function to increase proviral expression in latently infected cells. We do know that LRAs that have been tested are not highly effective because only a fraction of the intact proviruses can be induced in vitro with a single round of treatment, either in vivo or in vitro [11,78]. Second and third rounds (and maybe more) of LRA treatment in vitro induce production of virus from cells not induced in previous rounds. What changed in the cells from one round to the next? Is it the cells in each clone that express low levels of RNA that are inducible? In other words, do the LRAs produce a relatively high level of viral RNA expression only in the few percent of cells already making low levels of viral RNA? Several of these questions can answered with the available technology and we look forward to experimental answers to these questions.

## 11. Clonal Expansion out of Control: HIV in Lymphomas

**Figure 9:**
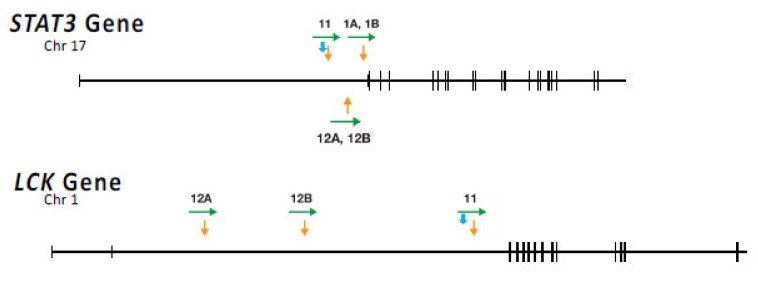
Locations of proviruses in T cell lymphomas. The orange arrows show the location of HIV proviruses in tumors from 3 different individuals (numbers 1, 11, and 12), with tumors obtained from different sites denoted as A and B [59]. The green arrows indicate the orientation of the integrated provirus. The transcribed portion of each gene is shown by the horizontal line, with short vertical lines denoting exons. The light blue arrow indicates the translational start site. Note that, in the case of lymphoma 12, both tumors had a provirus at exactly the same site in *STAT3*, but, although both tumors had a provirus in in *LCK*, the proviruses were in different sites. This pattern indicates that *STAT3* integration was followed by growth and spread of the tumor, which must have occurred before the two independent integrations into *LCK.* From [59].

HIV infection is associated with increased incidence of a number of cancers, many of which are caused by other opportunistic viruses, such as Epstein–Barr virus, Kaposi’s sarcoma herpesvirus, or human papillomavirus, which replicate much more efficiently in an immunocompromised individual. In contrast, there is also an increase in the incidence of non-Hodgkin’s B-cell lymphomas and, to a lesser extent, T cell lymphomas that has not been associated with an opportunistic infection [85,86]. As we have discussed, HIV integration can promote the expansion and/or survival of target cells by integration into one of a handful of genes. Although this process appears to be analogous to well-known models of oncogenesis by many other retroviruses, clones with proviruses in these genes are not cancers. Can HIV proviruses be directly involved in causing cancer? Recent evidence shows that some AIDS-related T cell lymphomas have clonal integration of HIV proviruses in the first intron of *STAT3* [59], a gene central to lymphocyte growth signaling, and one often mutated in non-AIDS lymphomas (Figure 9). In one particularly interesting example, the provirus is missing almost all of the 5′ LTR, including the transcription initiation site, and the 3′ LTR is driving high-level expression of *STAT3*. LTR- *STAT3* expression appears to have been aided by the expression of *tat*, not from the absent 5’ LTR, but rather from the upstream *STAT3* promoter. A number of tumor samples also have a second clonal provirus in a well-known tyrosine kinase oncogene, *LCK* (Figure 9), implying that misexpression of both genes is involved in the oncogenic process. In one case, tumor samples from two different locations in the same individual had a provirus at the identical site in *STAT3* but different sites in *LCK*. This observation shows that, at least in this case, integration into *STAT3* occurred before a second, independent integration into *LCK*. The data also strongly suggest that additional non-viral mutations are also important to the generation of a fully malignant cell. 

That provirus-driven misexpression of *STAT3* can promote the growth of T cells is also supported by an in vitro study, in which freshly isolated CD4+ T cells were infected with a non-replicating HIV vector and maintained, with occasional stimulation, for about 2 months. Integration site analysis revealed a relatively high level of expansion of cells with proviruses all in the same orientation as the *STAT3* gene [37]. However, in the lymphomas, the proviruses would be expected to induce the expression of the full length STAT3 protein and in the in vitro experiments, most, but not all of the proviruses would be expected to induce the expression of a slightly truncated form of STAT3. RNA analysis showed that the proviruses were also driving *STAT3* expression, in this case from the 5′ LTR promoter. Splicing from HIV donors produced fusion RNAs linked to an acceptor in the second, third, or fourth *STAT3* intron. Interestingly, although *STAT3* is a good target for integration both in vitro and ex vivo, it was not seen as one of the seven genes in which proviruses are associated with clonal expansion in vivo [37], nor were any of the seven genes in which a provirus is associated with clonal expansion in vivo identified in the in vitro experiments. 

The most frequent AIDS-associated lymphomas are of B cell, not T cell, origin. However, B lymphocytes are generally not considered to be targets for HIV infection. Given that B cell infection has been reported to occur in vitro, albeit with very low efficiency [87], and given the very large number of T cells infected every day, even a relatively inefficient infection of B cells could be enough to yield an occasional integration in *STAT3*, which is a good target, both in vitro and in vivo [37]. Further, unlike transformed CD4+ T cells, HIV-infected B cells would not be highly susceptible to killing by superinfection with HIV. In any case, there is a single published case report of an AIDS-related B cell lymphoma with a provirus in the same region of *STAT3* and in the same orientation as the gene, similar to the T cell lymphomas [59] and the in vitro data discussed above [88]. If HIV integration is found to be a frequent cause of lymphomas, it could suggest new avenues of treatment for this difficult and deadly disease.

## 12. Reprise

The importance of latently infected CD4+ T cells in preventing suppressive ART from curing HIV infection has been apparent for more than 20 years. It is only in the last seven years that we have come to appreciate the true nature of the reservoir (defined as the cells carrying latent proviruses capable of induction to yield infectious virus). We no longer view the reservoir as a static collection of resting CD4+ T cells infected at some time before ART and waiting for some stimulus related to ART cessation to start producing infectious virus. Rather, the reservoir is comprised primarily—perhaps entirely—of clones of cells that carry intact infectious proviruses, a small fraction of which appear to be producing low levels of virus at any one time. The reservoir has the following characteristics:

- The reservoir comprises a complex mixture of clones of that vary in size and composition among individuals.
- Clones arise from a very small fraction of the infected cells, some of which were infected very early (ca. 2–3 weeks or less) after an individual was infected.
- Clones can grow to a very large size within a few weeks, an observation incompatible with the formation of the reservoir by infection of resting cells.
- Whatever its mechanism(s), proviral latency must be maintained in the large majority of cells in each clone during both antigen-driven proliferation and homeostatic T-cell maintenance.
- Expansion of clones of infected cells to a size large enough to be detectable (10^4^ to 10^5^ cells) is, in a large majority of cases, caused by response to antigen. Homeostatic proliferation is unlikely to selectively drive the number of T cell generations needed to generate a large clone.
- Integration in one of seven oncogenes can lead to the misexpression of the surrounding gene, providing a selective advantage to the infected cell. This phenomenon, although striking, is limited to only about 2% of total unique integration events
- Overall, clonal expansion of infected cells, both during pretherapy and on ART, is independent of the site of integration or orientation of the provirus.
- With the possible exception of proviruses integrated in centromeric repeats, the available data strongly suggest that the position in the genome at which a provirus integrated has little or no effect on whether the provirus is expressed.
- In individuals on long-term ART, only a very small fraction of clones (2–5%) contain intact proviruses capable of producing infectious virus.
- Although both intact and defective proviruses seem to be expressed at similar low frequencies and seem to produce similarly low levels of RNA, there is no reason to assume that the mechanisms controlling their expression are the same. Therefore, shock and kill or other cure studies that use bulk DNA assays as the readout could miss an important effect on the small fraction of cells that carry intact, infectious proviruses.
- For at least some clones with intact proviruses, a very small fraction of the cells in the clone are constantly releasing sufficient virus to be detectable as persistent viremia using sensitive assays.
- At the moment, we have no clue as to what factors, or natural stimuli, lead to virus production in cells carrying latent proviruses in those living with HIV.
- There is no reason to think that ART discontinuation leads to induction of virus production. Rather, rebound viremia following ART discontinuation is almost certainly the result of infection and replication by the low level of virus present in most people at the time ART is stopped.
- It follows that careful quantitative measurement of the effects of candidate LRAs on low level persistent HIV, particularly in individuals who express high levels of viremia, may be the most effective way to assess their efficacy.

Curing HIV infection is one of the most sought after, yet elusive goals of current biomedical science. It is our fervent hope that a deeper understanding of the clonal structure of the HIV reservoir in people on fully suppressive antiviral therapy will clarify the problem and perhaps even point the way to an effective therapy. 

## Figures and Tables

**Table 1:** Genes in which proviruses can provide a selective advantage ^a^.

Gene Name	Unique Integration Sites in Genes on–ART(%of Total)	Integration Sites in PBMC	PBMC/on ART (Enrichment) ^b^	Enrichment Probability (Poisson)	Provirus Orientation: Same as Gene/Opposite	Orientation Probability (Binomial)	Positions of Selected Proviruses Relative to Protein Coding exons
All genes	25,731(100)	326,033	12.67(1.0)		ll,476/14,255	7.0 × 10^−60^	
*STAT5B*	268(1.0)	562	2.1(6.0)	1.4 × 10^−114^	197/71	3.5 × 10^−15^	Upstream
*BACH2*	98(.04)	132	1.3(8.7)	9.2 × 10^−06^	71/20	3.6 × 10^−08^	Upstream
*MKL2*	49(.02)	69	1.4(8.5)	5.7 × 10^−27^	40/6	1.6 × 10^−07^	In between
*MKL1*	85(.03)	331	3.9 (3.2)	3.5 × 10^−18^	53/30	7.6 × 10^−03^	In between
*IL2RB*	30(.011)	68	2.3(4.8)	1.8 × 10^−04^	17/9	8.4 × 10^−01^	Upstream
*MYB*	11(.004)	31	3.1(4.1)	2.3 × 10^−05^	10/0	9.8 × 10^−04^	In between
*POU2F1*	15(.006)	43	2.9(4.4)	7.5 × 10^−07^	10/5	1.5 × 10^−01^	Upstream
**Seven** **Gene Total**	**556 (2.2)**	**1236**	**2.2 (5.7)**		**400/156**		

^a^ From [37] ^b^ Enrichment factor (in parentheses): ratio of the number of integration sites on ART/sites in PBMC times 12.67.
